# State Transition Graph-Based Spatial–Temporal Attention Network for Cell-Level Mobile Traffic Prediction

**DOI:** 10.3390/s23239308

**Published:** 2023-11-21

**Authors:** Jianrun Shi, Leiyang Cui, Bo Gu, Bin Lyu, Shimin Gong

**Affiliations:** 1 School of Intelligent Systems Engineering, Shenzhen Campus of Sun Yat-Sen University, Shenzhen 518107, China; shijr5@mail2.sysu.edu.cn (J.S.); gubo@mail.sysu.edu.cn (B.G.); 2 Huawei Cloud, Shenzhen 518129, China; cuileiyang1@huawei.com; 3 Key Laboratory of Ministry of Education in Broadband Wireless Communication and Sensor Network Technology, Nanjing University of Posts and Telecommunications, Nanjing 210003, China; blyu@njupt.edu.cn; 4 Guangdong Provincial Key Laboratory of Fire Science and Intelligent Emergency Technology, Guangzhou 510006, China

**Keywords:** mobile traffic prediction, graph neural network, attention mechanism, long short-term memory

## Abstract

Mobile traffic prediction enables the efficient utilization of network resources and enhances user experience. In this paper, we propose a state transition graph-based spatial–temporal attention network (STG-STAN) for cell-level mobile traffic prediction, which is designed to exploit the underlying spatial–temporal dynamic information hidden in the historical mobile traffic data. Specifically, we first identify the semantic context information over different segments of the historical data by constructing the state transition graphs, which may reveal different patterns of random fluctuation. Then, based on the state transition graphs, a spatial attention extraction module using graph convolutional networks (GCNs) is designed to aggregate the spatial information of different nodes in the state transition graph. Moreover, a temporal extraction module is employed to capture the dynamic evolution and temporal correlation of the state transition graphs over time. Such a spatial–temporal attention network can be further integrated with a parallel long short-term memory (LSTM) module to improve the accuracy of mobile traffic prediction. Extensive experiments demonstrate that the STG-STAN can better exploit the spatial–temporal information hidden in the state transition graphs, achieving superior performance compared with several baselines.

## 1. Introduction

Mobile networks enable seamless connectivity and communication in a variety of application scenarios, such as cellular communication [1], Internet of Things [2], video streaming services [3], and vehicular communications [4]. The diversification of application scenarios leads to the rapid growth of the demand for mobile network resources. Consequently, there is a growing consensus on the utilization of artificial intelligence (AI) for self-optimizing network resource management. With recent advancements in data acquisition technologies, network operators can collect large amounts of relevant data to perform predictive analytics on network resources. This empowers network operators to make well-informed decisions, subsequently improving the overall capacity and reliability of mobile networks. Mobile traffic prediction is one of the critical works for resource scheduling [5]. It not only provides insights for base station deployment but also serves as an early warning system for sudden surges in mobile traffic demand. For example, based on mobile traffic prediction, network operators can design an adaptive sleeping strategy for cellular towers, which is one of the most energy-consuming components of mobile networks [6].

Despite the benefits, mobile traffic prediction is a highly challenging task. The patterns of mobile traffic are complex due to a variety of factors, such as diverse business requirements, user mobility, and social activities [7]. In urban areas, mobile traffic exhibits extensive spatial–temporal patterns. In the spatial domain, the complexity of mobile traffic usually stems from the functionality of regions. Mobile traffic within the same functional region often exhibits similar patterns, even if they may be geographically distant. Conversely, two neighboring regions may display significant differences in traffic patterns because of their distinct functionalities. In the temporal domain, the complexity of mobile traffic often arises from human activities. For instance, in residential areas, mobile traffic does not vary significantly between weekdays and weekends. However, in office areas, there is a noticeable drop in traffic demand during weekends.

Traditional machine learning models such as support vector regression (SVR) and random forest (RF) have been extensively employed for mobile traffic prediction. While these models are characterized by their lightweight nature and ease of training, their ability to capture complex patterns in mobile traffic is limited due to their relatively small parameter space. In recent years, because of the strong nonlinear modeling capabilities of deep learning (DL), there has been a lot of research applying DL techniques to mobile traffic prediction [8,9]. Most of the works employ graph-based structures to model the spatial dependencies of mobile traffic. After constructing graphs, convolutional neural networks (CNNs) and recurrent neural networks (RNNs) are often utilized to capture mobile traffic patterns in both spatial and temporal domains. Nevertheless, training a spatial–temporal prediction model based on DL typically requires large-scale urban mobile traffic data. In cases with limited or singular region data, like when deploying a new base station, these methods may not be effective. Consequently, performing traffic prediction using only cell-level data holds practical significance.

In this paper, we introduce a deep learning-based framework for cell-level mobile traffic prediction. Our goal is to effectively capture the underlying spatial–temporal patterns within a single cellular region. In contrast to existing approaches that utilize geographical graphs to model real-world spatial correlations, our approach involves the design of state transition graphs to capture long-term mobile traffic patterns. Notably, the state transition graph operates without the need for geographical location information. It constructs spatial graphs based on individual mobile traffic sequences to represent potential patterns. In particular, we divide mobile traffic data into distinct ranges to denote states. State transitions are represented as dynamic graphs, reflecting the evolving nature of mobile traffic over time. By constructing the state transition graph, we can capture spatial interdependencies among different nodes. Furthermore, this graph provides supplementary insights into traffic data patterns and reflects contextual information about the cellular region. For instance, the state transition graph for a cellular base station may reveal significant distinctions between weekdays and weekends.

Specifically, we propose a state transition graph-based spatial–temporal attention network (STG-STAN) for cell-level mobile traffic prediction, which leverages dynamic state transitions to exploit the spatial–temporal evolution of a single region’s traffic data. Firstly, we divide mobile traffic into different segments and construct a state transition graph for each segment. These graphs capture diverse patterns that help us understand the contextual information of the cellular region. Each node in the state transition graph represents a state, and the edges denote the transitions between states. Next, we employ graph convolutional networks (GCNs) to aggregate the information from the neighborhoods of each node within the state transition graph, which allows us to capture the spatial dependencies among the nodes. Moreover, in order to incorporate the global spatial correlations, we take into account the connections among all nodes in the graph and propose a node attention mechanism. The spatial representations obtained from the state transition graphs are then fed into RNNs to extract the temporal dependencies among the different state transition graphs. Such a spatial–temporal attention network is further integrated with a parallel long short-term memory (LSTM) branch to fuse multi-scale features in the mobile traffic for more accurate prediction. We summarize our contributions as follows:

- We propose a novel STG-STAN for predicting mobile traffic at the cell level, which leverages the dynamic evolution of underlying state transitions to achieve precise prediction.
- In the STG-STAN, we construct state transition graphs for capturing the spatial–temporal correlations presented in mobile traffic data. This approach enables the STG-STAN to effectively model the complex dependencies and interactions between different traffic states over time. Then, a parallel LSTM branch in the STG-STAN enhances the ability to capture both small-scale and large-scale temporal characteristics of mobile traffic.
- Extensive experiments demonstrate that the STG-STAN can better exploit the spatial–temporal information and achieve superior performance gain compared with typical baselines.

The remainder of this paper is organized as follows. We discuss related works in Section 2. We give preliminary observations in Section 3. The framework of the STG-STAN is detailed in Section 4. Finally, we present the experiment’s results in Section 5 and conclude the paper in Section 6.

## 2. Related Work

### 2.1. Cellular Traffic Prediction

The pattern of cellular traffic is complicated due to the spatial–temporal correlation, which is influenced by various factors, e.g., diverse user demands and user mobility. Traditional models that only consider temporal relationships perform poorly because they fail to capture spatial correlations. Additionally, due to the limited parameters of these models, they cannot well fit the non-linear pattern of mobile traffic. In order to extract spatial correlations, several spatial modeling methods have been proposed for city-level mobile traffic prediction. These approaches typically utilize geographical information to represent spatial data. Consequently, mobile traffic from two regions that are geographically adjacent is considered to exhibit higher levels of similarity. For example, by taking into account the decomposition of mobile traffic, the authors in [10] modeled the spatial–temporal dependencies of traffic using a graph representation. The authors in [11] treated mobile traffic data as images to extract the spatial and temporal correlations among different cells. The authors in [12] proposed a time-wise attention-aided convolutional neural network to capture the spatial–temporal correlation of mobile traffic, in which convolutional layers were employed for the purpose of capturing local feature maps.

Although using geographical locations for graph construction is intuitive, the performance of prediction models may be undermined because they may include neighboring areas that are irrelevant and have a detrimental effect. To address this problem, some studies considered dynamic connections among regions in the city. In this approach, the connection between different regions is not determined by physical distance, but rather by correlations of mobile traffic patterns, such as similarity. The authors in [13,14,15] utilized dynamic time warping as a method for measuring the similarity between the cellular traffic of two regions, thereby representing spatial information. The authors in [16] considered the correlations of non-local areas, which exhibit relevant traffic patterns. Then, the spatial information was combined with different periodic features to achieve better performance.

After spatial modeling, the spatial–temporal information of mobile traffic is generally constructed using CNN, GNN, and RNN. For instance, the authors in [17] adopted CNN layers to fuse the local spatial information and LSTM layers to capture temporal information. The external impact factors were also considered and modeled by CNN layers. The authors in [18] combined GCN and gated recurrent unit to learn the spatial–temporal dependency of traffic. The authors in [8] adopted an attention mechanism to capture diverse global spatial–temporal dependencies in cellular networks. The local spatial–temporal dependencies were extracted by adopting dense convolution. In order to obtain the temporal pattern, the authors in [19] proposed three temporal components to model different periodic patterns of mobile traffic. The spatial–temporal correlation of mobile traffic was then captured by utilizing the attention mechanism and convolution operation.

The aforementioned studies deal with large-scale city-level mobile traffic prediction, where the exploration of spatial patterns typically spans across multiple cellular regions. In contrast, this work focuses on cell-level mobile traffic prediction, aiming to capture effective spatial–temporal patterns from individual traffic sequences to enhance prediction accuracy. In addition to cellular traffic prediction, other areas of traffic forecasting, such as traffic speed prediction, also widely employ spatial–temporal attention networks. For instance, the author in [20] utilized the graph attention network and an LSTM network to capture spatial–temporal features. Although spatial–temporal attention networks have been widely studied, to the best of our knowledge, our work is the first to apply them to cell-level mobile traffic prediction. Furthermore, we propose state transition graphs to model the long-term patterns of mobile traffic in individual regions, making it feasible to employ spatial–temporal feature extraction techniques for cell-level mobile traffic prediction.

### 2.2. Dynamic Graph Neural Networks

Graph neural networks have been widely applied to solve a variety of problems, ranging from link prediction [21], node classification [22], graph generation [23], recommendation systems [24], and so on. In many real-world scenarios, graph data change over time and modeling dynamic graphs has thus become an important research area in recent years. The topology and properties of dynamic graphs change over time, making it challenging to capture and analyze the underlying patterns and dynamics. To address the problems, researchers have proposed several approaches for modeling dynamic graphs, which typically use GNNs to capture graph information and adopt RNNs to learn the dynamics. The authors in [25] combined static graph representation methods and an RNN to construct dynamic graph representations. Gated GNNs and LSTM were designed with the aim of maintaining the structure of a graph and capturing its temporal behavior. The authors in [26] modeled the dynamic information as the graph evolution, including the update and propagation of graph node information. Upon the introduction of a new edge, the creation sequential information of edges and the time intervals between interactions were adopted to update node information. Considering that some nodes may not be present all the time, the authors in [27] used RNNs to evolve the network parameters of GNNs, where only the RNN parameters were trained in the proposed method. A spatial–temporal self-attention mechanism was proposed in [28] for dynamic node representations. In order to capture multiple types of graph evolution, multi-head attention was used in both spatial and temporal layers. The authors in [29] studied time series event prediction by constructing dynamic graphs. An evolutionary state graph network model was proposed to capture the node-level and graph-level evolution.

For traffic forecasting tasks, dynamic graph neural networks are typically employed to model the dynamic relationships among nodes, or to extract evolving node information across different time steps. The authors in [30] integrated a GCN and gated recurrent unit to capture topological structure and temporal features of road networks. The nodes on the graph corresponded to roads, and the edges denoted the connections between roads. The authors in [31] proposed a graph generation module to dynamically integrate geographic proximity and spatial heterogeneity information across different times. By generating dynamic adjacency matrixes, the dynamic characteristics of road networks could be modeled. The authors in [32] proposed an adjacency learning mechanism to capture the dependency among traffic sensors. The traffic data correlations were captured through a dynamic graph construction algorithm.

Our proposed scheme leverages a hybrid approach, combining a GNN and RNN to effectively capture spatial–temporal traffic characteristics. However, it is worth noting that GCN layers are inherently designed to incorporate future states into the current state, without explicitly considering the exploration of global correlations among already integrated features. Unlike the works above, we introduce a novel node self-attention mechanism. This mechanism aims to enhance the model’s ability to capture global spatial connections among nodes following the feature aggregation process.

## 3. Preliminary Observation and Analysis

**Figure 1:**
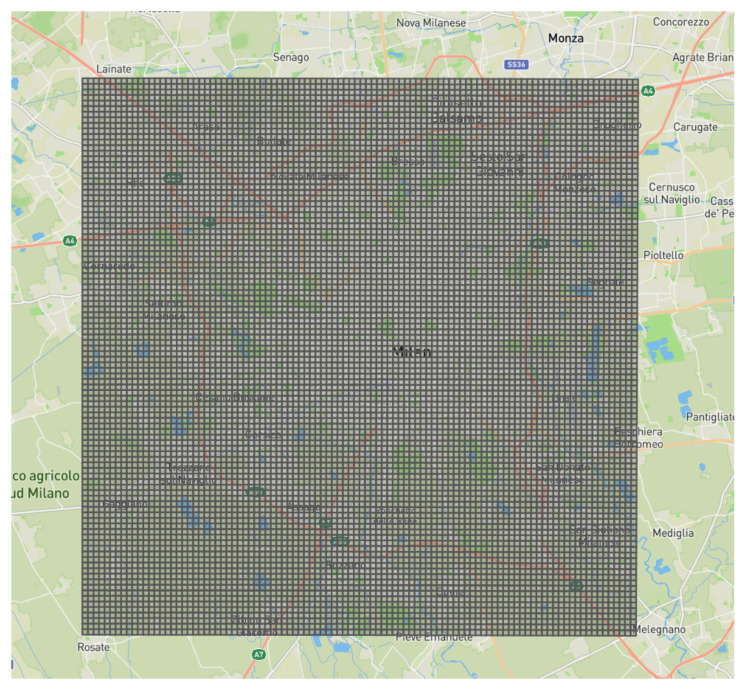
The area of Milan is composed of a grid overlay of 10,000.

To further illustrate the temporal dynamics of mobile traffic, we conduct an analysis on a real-world citywide mobile traffic dataset [33]. This dataset was published by Telecom Italia and contains three types of mobile traffic records, i.e., short message service (SMS), call service, and Internet service, over the city of Milan. The records are aggregated in a 100 × 100 partition and the size of each square is about 235 × 235 m. Figure 1 shows the Milan urban area by using a grid. The mobile traffic was collected from 1 November 2013 to 1 January 2014 with an interval of 10 min.

**Figure 2:**
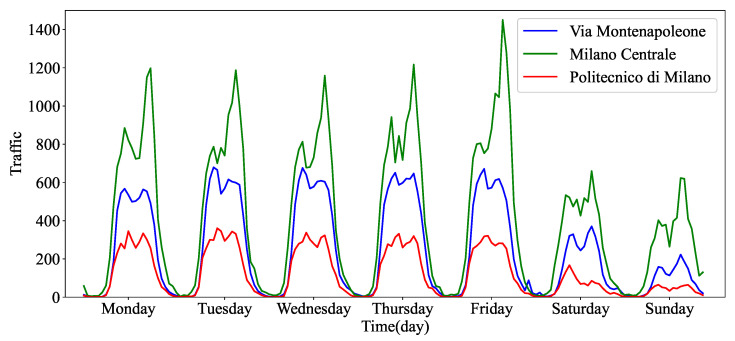
The temporal characteristic of mobile traffic from three regions.

Figure 2 shows visualized records of one week of mobile traffic from three distinct regions within a city. The horizontal axis represents the time interval index on an daily scale, while the vertical axis denotes the number of telecommunication events occurring within a specific region. We choose three functionally different areas, including Via Montenapoleone, Milano Centrale, and Politecnico di Milano, which represent commercial, transportation, and educational zones, respectively. From Figure 2, several observations can be found. Firstly, there is a clear periodicity in the fluctuations of mobile traffic throughout the week. Each day exhibits a consistent pattern with two traffic peaks. These peaks coincide with the morning and evening rush hours, corresponding to people commuting to and from work. Secondly, there are significant disparities in mobile traffic patterns between weekdays and weekends. During the workweek, typically from Monday to Friday, the demand for mobile traffic is noticeably higher than that during the weekends. In addition, there are small-scale fluctuations in mobile traffic. These fluctuations are inherently present due to short-term variations in demand, which can be influenced by various factors such as special events, weather conditions, and local activities. Lastly, it is essential to acknowledge the heterogeneity in mobile traffic across the functional areas. For instance, Milano Centrale stands out as having notably higher nighttime traffic demand compared with Via Montenapoleone and Politecnico di Milano. This divergence underscores the influence of the specific characteristics and functions of each region on mobile networks.

**Figure 3:**
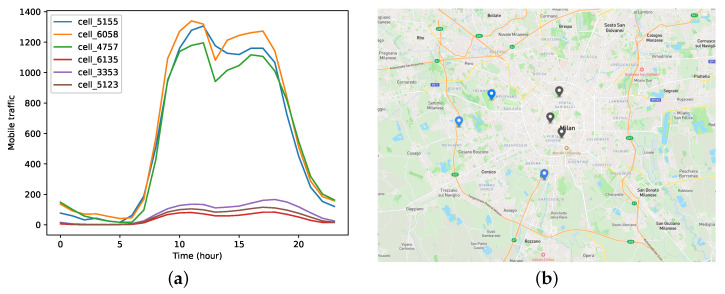
The spatial analysis of mobile traffic. (**a**) The mobile traffic of six regions in two clusters. (**b**) The locations of the six regions in Milan.

Furthermore, we conduct a clustering analysis on different regions in Milan to demonstrate the spatial correlation of mobile traffic. Specifically, we aggregate the traffic data at an hourly granularity. Subsequently, we process the data over a span of 62 days by calculating the average 24 h mobile traffic, which serves as the feature for clustering. Figure 3a displays the mobile traffic patterns of six regions in two clusters. It is evident that there exists similarity among the mobile traffic patterns. Additionally, we mark the specific locations of these six regions on the map, as shown in Figure 3b, with the regions of the two categories differentiated by blue and gray colors. The results indicate that despite the absence of geographical adjacency between different regions, there is still a correlation in mobile traffic patterns, which implies that knowledge transfer can be conducted based on the spatial correlation between regions. In addition, it can be observed that regions near the city center and those far from the city center exhibit significant differences in mobile traffic patterns. This discrepancy is generally determined by the density of population aggregation.

## 4. STG-STAN Framework for Mobile Traffic Prediction

Based on the analysis above, we propose a novel state transition graph-based spatial–temporal attention network for mobile traffic prediction. In this section, we begin by providing an overview of the STG-STAN. Then, we present the design of the STG-STAN in detail.

### 4.1. Overview of the STG-STAN

Let $x_{t}$ denote the measurement of mobile traffic at time *t* and $X=\{x_{t-\tau +1},x_{t-\tau +2},\cdots ,x_{t}\}$$\in R^{\tau }$ denote a set of historical measurements at time *t*, where τ is the size of the moving window. In this work, we focus on the one-step-ahead prediction. The mobile traffic prediction problem can be described as follows:

(1) $${\hat{x}}_{t+1}=f\left(x_{t-\tau +1},x_{t-\tau +2},\cdots ,x_{t}\right)$$

where ${\hat{x}}_{t+1}$ is the prediction and f(·) is a nonlinear mapping function, which is learned by deep neural networks.

**Figure 4:**
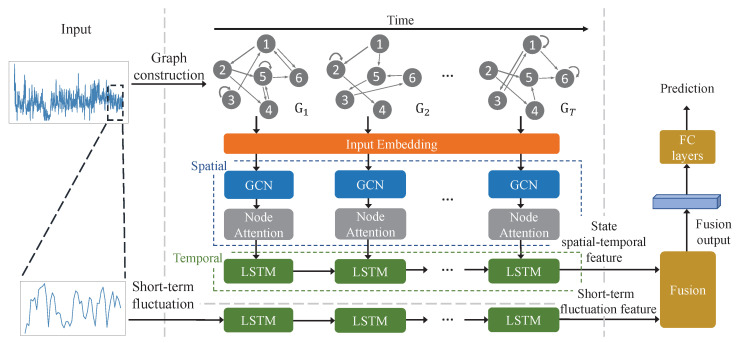
STG-STAN framework for mobile traffic prediction.

The basic idea of the STG-STAN is to partition mobile traffic data into states and use the transition patterns between states to characterize the long-term fluctuation characteristics of mobile traffic. By constructing state transition graphs, more comprehensive feature representations can be obtained by leveraging deep neural networks to explore spatial and temporal information, thereby enhancing the prediction performance. The structure of our proposed STG-STAN comprises three main components, as depicted in Figure 4. The first component is the spatial–temporal attention network for the state transition graphs, which is used to capture the spatial–temporal dependencies of mobile traffic data. Specifically, the spatial–temporal attention network includes two modules, i.e., the spatial attention module and the temporal evolution module. The former employs GNNs and a self-attention mechanism to extract structural information from the state transition graph. The latter adopts LSTM networks to capture the temporal evolution of nodes over different state transition graphs. The second component is an independent LSTM branch that captures the short-term dynamic characteristics of the traffic data. The third component is the fusion module, which combines the outputs from the first and the second components using a weighted feature fusion mechanism to generate the final prediction output.

Unlike traditional ML-based and DL-based models, which omit state transition information, the STG-STAN leverages spatial–temporal dependencies and features hidden in cell-level mobile traffic. The overall process of our proposed method is divided into four steps, which are briefly described as follows:

(1) In step 1, we model traffic changes over time as state transitions and construct dynamic graphs to represent the spatial–temporal correlations of traffic states.
(2) In step 2, the node embeddings of dynamic graphs are fed into the spatial attention module. In this module, GCNs and node attention layers are employed to extract local and global spatial correlations between nodes. Specifically, local structural correlation is based on the aggregation of information between adjacent nodes, while global spatial information is captured through the state transition similarity on the whole graph. The node vectors containing spatial information are then fed into the temporal information evolution module to capture the dynamic temporal patterns of each node.
(3) In step 3, considering the short-term fluctuation characteristics of mobile traffic, we adopt an LSTM branch to extract small-scale temporal patterns of traffic data.
(4) In step 4, the prediction is obtained by fusing the node vector output and the parallel LSTM output. Through the extraction of the spatial–temporal state correlations and short-term fluctuation characteristics, more abundant mobile traffic patterns can be obtained.

### 4.2. Graph Representation for State Transition

In order to model the correlations between traffic states, we transform the traffic time series into state transition graphs, which may provide additional semantic information to predict future trends. The spatial correlation in the graph can also be exploited as a representation of the semantic information. In this subsection, we introduce the graph representation for state transition in detail.

#### 4.2.1. Segmentation of the Traffic Time Series

Given the input mobile traffic data X, we perform segmentation based on statistical properties, i.e., the mean and variance. These segments serve as the basis for constructing state transition graphs denoted as $G_{1},G_{2},\cdots ,G_{T}$. We employ the existing offline change point detection methods for time series segmentation [34]. Based on the analysis in Section 3, there are three main reasons for segmentation. Firstly, segmentation is advantageous in discerning periodic patterns within the mobile traffic data. After segmentation, we can effectively differentiate and model daily patterns separately by employing state transition graphs. Secondly, segmentation enables a more precise characterization of the heterogeneity exhibited by different regions, providing a level of interpretability. Thirdly, as the constructed graphs exhibit dynamic evolution following segmentation, we can leverage dynamic graphs to model the evolving characteristics of mobile traffic, distinctly capturing features separated from small-scale fluctuations.

#### 4.2.2. Constructing State Transition Graphs

**Figure 5:**
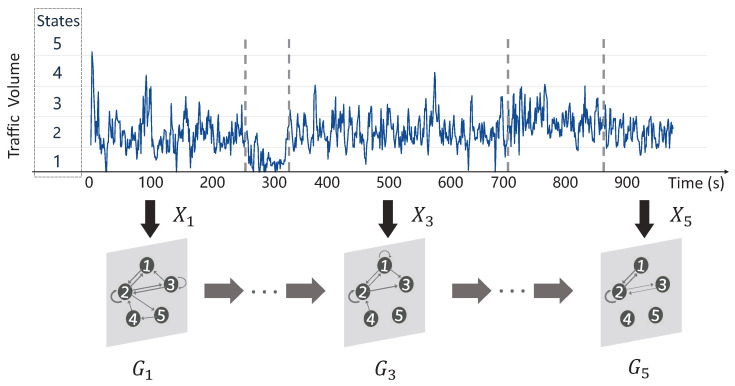
An illustrative example of state transition graphs with 5 states.

The construction of a state transition graph within each segment relies on the dynamic properties of the traffic data. Before constructing the graph, we perform equal quantization of the traffic measurement data into *N* bins and map each measurement sample $x_{t}$ into one of bins. We define dynamic graphs as a sequence of state transition graphs, denoted as $G=\{G_{1},G_{2},\cdots ,G_{T}\}$, where each $G_{k}=<V_{k},E_{k}>$ represents a weighted directed graph. In the graph, the set of vertices and edges are denoted as $V_{k}$ and $E_{k}$, respectively. Each node $v_{i}^{k}\in V$ in the graph $G_{k}$ indicates state-*i* and each edge $e_{ij}^{k}$ denotes the state transition from state-*i* to state-*j*. We use an adjacency matrix $A_{k}={\left\{a_{ij}^{k}\right\}}_{i,j\in \{1,2,\cdots ,N\}}$ to characterize the state transition, where $a_{ij}^{k}\in A_{k}$ represents the transition probability from state-*i* to state-*j* within the *k*-th segment. Specifically, $a_{ij}^{k}$ is evaluated as

(2) $$a_{ij}^{k}=\frac{c_{ij}^{k}}{l^{k}-1}$$

where $c_{ij}^{k}$ denotes the number of times when the traffic measurement transits from state-*i* to state-*j*, and $l^{k}$ denotes the length of segment-*k*. An illustrative example of constructing the dynamic state transition graphs is shown in Figure 5.

### 4.3. Spatial–Temporal Graph Attention Network

#### 4.3.1. Spatial Attention Module

To leverage the state transition information encoded in the graphs, we propose a spatial attention module that integrates GNN layers and a node attention mechanism. The GNN layers are utilized to capture local spatial information within the graph structure, while the node attention mechanism dynamically calculates similarity, allowing the model to prioritize the most relevant information.

Initially, we aggregate neighbor information into nodes using transition probabilities along the edges. To be specific, we input snapshots of the state transition graph into multiple independent GNNs. Before the aggregation operation, we apply an embedding layer to produce the initial feature for each node, resulting in a *d*-dimensional vector. The parameters of the embedding layer are updated during training. The input to the GNN layers consists of a set of node representations $\left\{v_{i}\in R^{d},\forall i\in V\right\}$, and the output yields a new set of node representations $\left\{z_{i}\in R^{D},\forall i\in V\right\}$, each possessing *D* dimensions that capture local spatial information. We employ a GCN in the spatial domain to capture local structural information. Spatial domain GCNs extend the concept of traditional GCNs, originally designed for graph-structured data, to effectively incorporate spatial information. Let $h_{i}^{\left(l\right)}\in R^{D}$ denote the feature vector of node-*i* in the *l*-th layer and then $h_{i}^{\left(l+1\right)}\in R^{D}$ can be computed as follows:

(3) $$h_{i}^{\left(l+1\right)}=\sigma \left(\sum_{j\in N(i)}a_{ij}h_{j}^{\left(l\right)}W_{0}^{\left(l\right)}+h_{i}^{\left(l\right)}W_{1}^{\left(l\right)}+b^{\left(l\right)}\right)$$

where N(i) represents the set of neighbors to node *i*. The parameters $W_{0}^{\left(l\right)}\in R^{D\times D}$ and $W_{1}^{\left(l\right)}\in R^{D\times D}$ denote the trainable weights in the *l*-th layer and $b^{\left(l\right)}\in R^{D}$ is the trainable bias in the *l*-th layer. Note that $a_{ij}$ is the weight of edge (i,j).

**Figure 6:**
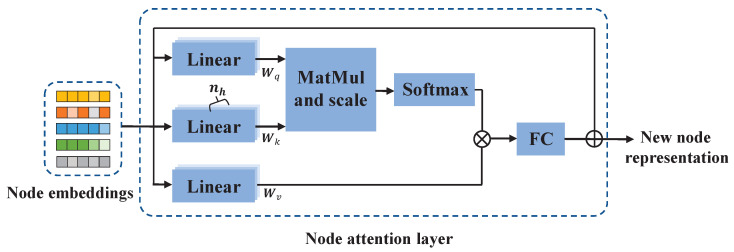
The structure of the spatial node attention layer.

In the state transition graph, the GNN layer integrates future mobile traffic states into the current state by considering the weights of the edges and node embeddings. However, such local spatial correlations are insufficient to represent the dynamic connection between nodes. To further extract the global spatial correlations, we propose a node attention mechanism. The structure of the spatial node attention layer is shown in Figure 6.

We denote the input and the new node representations as matrices $Z\in R^{N\times D}$ and $M\in R^{N\times F}$, respectively, where *F* is the dimension of new representations. The self-attention layer performs a linear projection of Z into multiple projection spaces by using three learnable matrices, $W_{q}\in R^{D\times F/n_{h}}$, $W_{k}\in R^{D\times F/n_{h}}$, and $W_{v}\in R^{D\times F/n_{h}}$, where $n_{h}$ is the number of attention heads. In each attention head, we employ the scaled dot-product attention mechanism [35] to calculate the similarity scores between nodes. The update of the node representations is calculated as follows:

(4) $$\begin{array}{cc} \; & c_{ij}=\frac{1}{\sqrt{F}}{\left(ZW_{q}{\left(ZW_{k}\right)}^{T}\right)}_{ij} \end{array}$$

(5) $$\begin{array}{cc} \; & \alpha_{i,j}=\frac{\exp(c_{i,j})}{\sum_{j=1}^{N}\exp\left(c_{i,j}\right)} \end{array}$$

(6) $$\begin{array}{cc} \; & M=\alpha ZW_{v} \end{array}$$

where $\alpha \in R^{N\times N}$ is the attention matrix and $\alpha_{i,j}$ indicates the importance of node *j* to node *i*. $M={\left\{m_{i}\right\}}_{i\in \{1,2,\cdots ,N\}}\in R^{N\times F/n_{h}}$ denotes the new representations.

We then concatenate the outputs from different attention heads and feed them into a fully connected feed-forward network, followed by a residual connection and normalization:

(7) $$\begin{array}{cc} \; & M^{\prime }=Concat\left(M_{1},M_{2},\cdots ,M_{n_{h}}\right) \end{array}$$

(8) $$\begin{array}{cc} \; & M=LayerNorm(Relu\left(M^{\prime }W_{fc}+b_{fc}\right)+M^{\prime }) \end{array}$$

where $W_{fc}\in R^{F\times F}$ denotes the learnable linear projection matrix and $b_{fc}\in R^{N\times F}$ is the bias.

#### 4.3.2. Temporal Evolution Module

**Figure 7:**
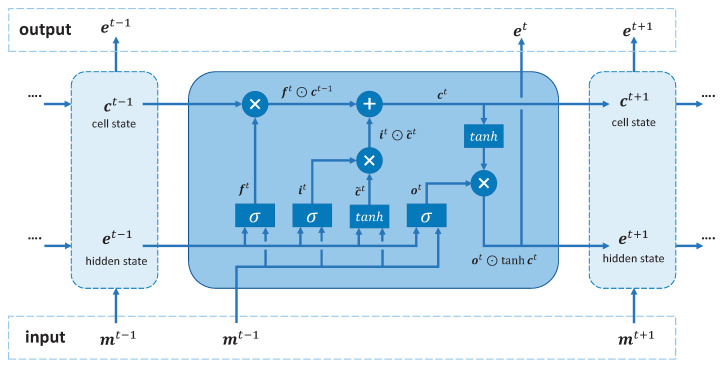
The structure of LSTM unit.

Given the node representations of each graph, we use an LSTM encoder to capture the temporal patterns of nodes among all graphs. LSTM consists of memory cells, input gates, output gates, and forget gates, which work together to selectively store or discard information, allowing the model to maintain long-term dependencies. The structure of the LSTM unit is shown in Figure 7. Specifically, the temporal module feeds the node representations $\left\{m_{i}^{1},m_{i}^{2},\cdots ,m_{i}^{T}\right\},m_{i}^{t}\in R^{F}$ into LSTM and generates the final embeddings $\left\{e_{i}^{1},e_{i}^{2},\cdots ,e_{i}^{T}\right\},e_{i}^{t}\in R^{F^{\prime }}$. The hidden state of each node at time *t* is computed as follows:

(9) $$\begin{array}{cc} \; & i^{t}=\sigma \left(W_{i}m^{t}+U_{i}e^{t-1}+b_{i}\right) \end{array}$$

(10) $$\begin{array}{cc} \; & f^{t}=\sigma \left(W_{f}m^{t}+U_{f}e^{t-1}+b_{f}\right) \end{array}$$

(11) $$\begin{array}{cc} \; & o^{t}=\sigma \left(W_{o}m^{t}+U_{o}e^{t-1}+b_{o}\right) \end{array}$$

(12) $$\begin{array}{cc} \; & {\tilde{c}}^{t}=\tanh\left(W_{c}m^{t}+U_{c}e^{t-1}+b_{c}\right) \end{array}$$

(13) $$\begin{array}{cc} \; & c^{t}=i^{t}⊙{\tilde{c}}^{t}+f^{t}⊙c^{t-1} \end{array}$$

(14) $$\begin{array}{cc} \; & e^{t}=o^{t}⊙\tanh\left(c^{t}\right) \end{array}$$

where $i^{t}$, $f^{t}$, and $o^{t}$ are the input gate, forget gate, and output gate, respectively. σ is a sigmoid function, and ⊙ is the element-wise product. To reduce computational complexity, we set the same LSTM parameters for each node. Let $s^{t}$ denote the traffic state at time *t*. We select the hidden vector $e_{s^{t}}\in R^{F^{\prime }}$ of state $s^{t}$ at time step *T* as the output.

### 4.4. Independent LSTM Branch and Fusion Output

According to the observation in Section 3, mobile traffic may experience short-term fluctuations, which can be caused by factors such as a sudden surge in user access to base stations, temporary obstructions due to buildings, and weather conditions. Therefore, it is crucial to accurately model the short-term evolution of mobile traffic, as relying solely on periodic patterns would overlook the valuable information contained in small-scale traffic patterns. To overcome the issue, an independent LSTM branch is adopted to model the short-term fluctuation characteristics of mobile traffic. Specifically, we feed the traffic data $\left\{x_{t-\tau^{\prime }},x_{t-\tau^{\prime }+1},\cdots ,x_{t}\right\}$ into an independent LSTM branch and generate the non-linear dynamic features. This module employs a much smaller window size $\tau^{\prime }$ ($\tau^{\prime }\ll \tau$) to effectively capture the local patterns of the traffic data, which provides a more robust and accurate representation of mobile traffic. The output of the LSTM branch is the hidden vector at time *t*, denoted as $p^{t}$.

At last, we employ a fusion module to integrate the outputs from both the spatial–temporal attention network and the independent LSTM branch, thereby enhancing the ability to capture multi-scale features. In particular, the spatial–temporal attention network captures the long-term dependencies in the time series, while the independent LSTM branch focuses on the short-term dynamics. In the fusion module, two trainable weight matrices are applied to map two features into projection spaces with the same dimensionality. Then, the final prediction is obtained as follows by feeding them to the fully connected layers.

(15) $${\hat{x}}_{t+1}=\sigma \left(\left(e_{s^{t}}W_{e}+p^{t}W_{p}\right)W_{fc}+b_{fc}\right)$$

where $W_{e}$, $W_{p}$, and $W_{fc}$ denote the trainable weights; $b_{fc}$ is the bias; and σ is the nonlinear activation function. Our model is trained in an end-to-end manner and we use mean squared error (MSE) as the loss function in model training, defined as $F_{loss}=\frac{1}{L}\sum_{t=1}^{L}{\left(x_{t}-{\hat{x}}_{t}\right)}^{2}$, where *L* is the total amount of training data, and $x_{t}$ is the ground truth at time *t*.

## 5. Experiments

In this section, we present a comprehensive analysis of our proposed approach by conducting extensive experiments on two real-world mobile traffic datasets. These experiments are aimed at verifying the efficacy of our methodology. In addition to the Milan dataset discussed in Section 3, we also leverage a dataset sourced from Trentino [33], a province in Italy. The Trentino dataset is structured as a grid consisting of 6575 squares and contains three types of mobile traffic. Importantly, it shares the same time span and time intervals as the Milan dataset, ensuring a consistent and meaningful comparison.

Firstly, we provide the experimental configuration, including key parameter settings, baselines, and performance metrics. Then, we present and analyze the experimental results of the STG-STAN from multiple perspectives. Finally, an ablation study is also conducted to validate the effectiveness of the key components in the proposed model.

### 5.1. Experimental Configuration

#### 5.1.1. Parameter Settings

As we mentioned before, the STG-STAN is designed for cell-level mobile traffic prediction. However, we can still utilize mobile traffic data from multiple regions to train the STG-STAN. Compared with the single-region model training approach, multi-region experiments involve solely increasing the number of training samples. This approach can provide a better basis for comparing the actual performance of different models, as the size of the dataset plays a crucial role in deep learning models. In this work, we evaluate the performance of models using both single-region and multi-region traffic data. In single-region experiments, we utilize data from a single cellular area for both model training and testing. To conduct these experiments, we randomly select 1000 regions in the Milan and the Trentino dataset. The final results are then derived as the average performance across these 1000 experiments. For multi-region experiments, our approach involves using traffic data from 20 randomly chosen regions for each individual model training and testing. We repeat this process three times to obtain averaged performance metrics. We rescale the data to a zero mean and unit variance during training. For evaluation, we anti-standardize the predicted values. A sliding window approach is employed to generate samples. The proportions for training, validation, and testing are 65%, 15%, and 20%, respectively.

Hyperparameters are tuned via grid search. For the spatial–temporal graph attention network, we set the number and the hidden dimension of GCN layers to 1 and 32, respectively, while the number of node attention heads is set to two. For the parallel LSTM branch, we choose the hidden states from {64, 128, 256} and the number of layers from {1, 2, 3}, respectively. The batch size is set to 32 for the STG-STAN, with an initial learning rate at 0.001 that is decayed by 0.5 every five epochs. The window sizes for the spatial attention module and the parallel LSTM branch are 168 and 20, respectively. All experiments are conducted with Tesla P4 GPU on an Ubuntu 18.04 Linux operating system with PyTorch 1.10.0.

#### 5.1.2. Baselines

To verify the performance of our proposed model, we compare it with two types of baselines, including non-spatial–temporal-based methods and spatial–temporal-based methods. The non-spatio–temporal-based methods take input from only a single cellular region:

- SVR: SVR is a supervised learning algorithm based on the principles of support vector machines. Its goal is to find the optimal hyperplane that maximizes the margin while ensuring that a predefined level of error is allowed for some data points.
- RF: RF is a machine learning algorithm that builds multiple decision trees and combines their predictions to reduce overfitting. Each tree is trained on a random subset of the data and the features, which helps to decorrelate the trees and make the model more robust.
- LSTM: long short-term memory (LSTM) can handle the vanishing gradient problem by using a gating mechanism, which has been widely applied in mobile traffic prediction.

The spatio–temporal-based methods take inputs from multiple cellular regions and concurrently predict the mobile traffic of multiple regions:

- CNN-LSTM [36]: CNN-LSTM is a hybrid neural network architecture that combines a CNN and LSTM to effectively extract spatial–temporal feature from mobile traffic.
- DenseNet [11]: DenseNet (Densely Connected CNN) is a DL-based model which utilizes a dense CNN to capture both the spatial and temporal dependence of mobile traffic.

#### 5.1.3. Performance Metrics

Three performance metrics are used to evaluate our model, including the root mean square error (RMSE), mean absolute error (MAE), and coefficient of determination (R2). The definitions of these metrics are as follows:

(16) $$\begin{array}{cc} \; & \mathrm{RMSE}=\sqrt{\frac{1}{N}\sum_{i=1}^{N}{\left(\hat{y_{i}}-y_{i}\right)}^{2}} \end{array}$$

(17) $$\begin{array}{cc} \; & \mathrm{MAE}=\frac{1}{N}\sum_{i=1}^{N}|\hat{y_{i}}-y_{i}| \end{array}$$

(18) $$\begin{array}{cc} \; & R2=1-\frac{\sum_{i=1}^{N}{\left(\hat{y_{i}}-y_{i}\right)}^{2}}{\sum_{i=1}^{N}{\left(y_{i}-\bar{y}\right)}^{2}} \end{array}$$

where $y_{i}$ is the ground truth, $\bar{y}$ is the mean of the observed data, $\hat{y_{i}}$ is the predicted value, and *N* is the total number of samples. MAE and RMSE indicate the prediction error of the model and R2 represents the correlation between the prediction and the ground truth. For MAE and RMSE, lower values are better, while for R2 higher values are better.

### 5.2. Experimental Results

#### 5.2.1. Performance Comparison of Models

Table 1 presents the evaluation results of various methods on mobile traffic. We evaluate the models using three kind of mobile traffic in Milan and Trentino. For the single-region data (*n* = 1), ML-based models demonstrate superior performance, especially the RF model. The results are reasonable since, compared with deep learning models, which require abundant training data, ML-based models have a smaller parameter space. Consequently, they can achieve satisfactory performance without the need for vast amounts of data.

When sufficient training data are available, as evidenced by the multi-region experimental results (*n* = 20), DL-based models demonstrate significantly better performance. The traffic on cellular networks displays apparent periodicity, which allows the DL-based models to learn and capture various traffic fluctuation characteristics. The STG-STAN outperforms all the baselines for all kinds of mobile traffic. The STG-STAN can effectively construct and capture the transition information of mobile traffic using state graphs and combine it with the small-scale features of the parallel LSTM branch to achieve better traffic prediction. Note that for a fair comparison, in the multi-region experiments, we concatenate the mobile traffic based on cell index, forming a 4×5×τ input vector for both CNN-LSTM and DenseNet, which are two spatial–temporal-based methods. We can observe that both CNN-LSTM and DenseNet demonstrate mediocre performances. This is attributed to their feature extraction process being reliant on large-scale data inputs. As a result, when data are limited to only a few regions, the performance of these models is significantly decreased.

**Figure 8:**
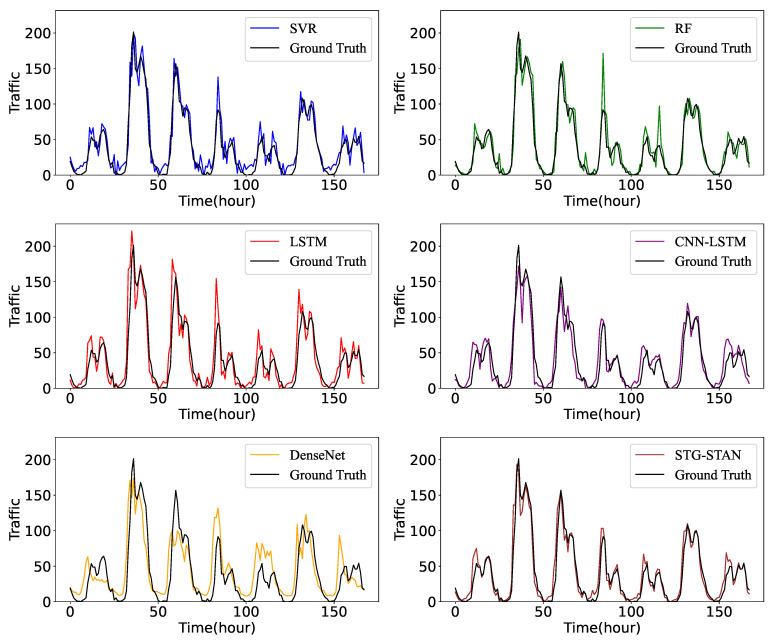
Prediction of cellular traffic in a cell for one week.

To better illustrate the performance differences among various models, we randomly select a region from the Milan dataset and visualize the ground truth of mobile traffic for one week and the predictions generated by various models, as depicted in Figure 8. Note that the predictions are generated by the models trained using multi-region traffic data. We can clearly see that the STG-STAN outperforms all the baselines, while it is difficult for ML-based models to fit complex cellular traffic. For instance, the predicted values of SVR exhibit significant deviations from the corresponding ground truth. The STG-STAN can effectively track the trend of traffic and make accurate predictions. For instance, the STG-STAN accurately predicts two traffic peaks after index 130, while other models fail to fit them. Compared with other models that only consider temporal changes, the STG-STAN can capture spatial information through dynamic state transition graphs and fuse temporal–spatial information, thus achieving the best performance.

**Figure 9:**
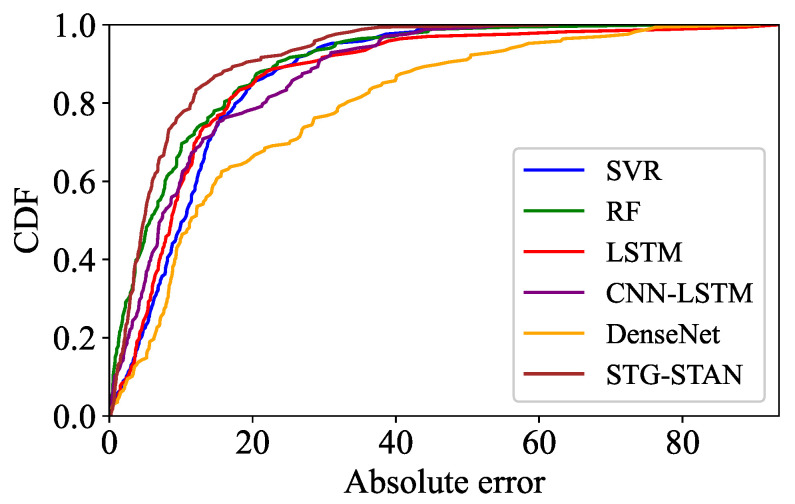
The prediction errors of different models.

We also compare the prediction errors of different models in the region depicted in Figure 8 using a CDF plot, as shown in Figure 9. It is evident that the overall prediction error of the STG-STAN (brown) is lower than that of the baselines, with approximately 80% of the prediction errors being below 10. These results indicate that the STG-STAN achieves better prediction performance than the baselines. This can be attributed to the outstanding spatial–temporal information mining ability of the STG-STAN. Compared with other models that only consider temporal changes, the STG-STAN can capture spatial information through dynamic state graphs and fuse temporal–spatial information to achieve accurate predictions.

Furthermore, we conduct experiments to analyze the impact of the number of states (*N*) in the STG-STAN, as presented in Table 2. In the Milan dataset, when *N* equals 15, 15, and 10 for SMS, call, and Internet traffic, respectively, the STG-STAN demonstrates the best performance, while in the Trento dataset, the STG-STAN performs best when *N* equals 100, 15, and 5 for SMS, call, and Internet traffic, respectively. Both excessively large and small values of *N* result in a decline in the model’s performance. When *N* is too small, the construction of traffic states becomes too coarse, potentially causing mobile traffic to remain in a single state, thereby impeding the extraction of valuable state evolution information. On the other hand, when *N* is too large, many irrelevant mobile traffic states may interfere with the effective identification of state transitions, thereby weakening the model’s performance. Striking the right balance in choosing *N* is essential to achieve optimal performance of the STG-STAN.

#### 5.2.2. Model Efficiency

Table 3 provides a comprehensive comparison of model efficiency, assessed in terms of parameters, training time, and inference time. Note that the experiments are conducted with the Milan dataset. The results highlight that ML-based models exhibit a relatively lightweight profile. In contrast, DL-based models come with a larger number of trainable parameters, resulting in increased resource requirements in terms of both space and time. However, they excel in modeling the intricate and dynamic characteristics of mobile traffic, offering superior performances. Furthermore, Table 3 emphasizes that the STG-STAN model incurs the highest computational cost among the models under examination. This trade-off is justifiable given the significant performance enhancements it delivers. Importantly, despite the relatively high computational cost, the inference time for the STG-STAN is a mere 1.85 s, underscoring its practical feasibility for deployment.

#### 5.2.3. Ablation Studies

To analyze the effectiveness of the node attention mechanism and the integration of multiple temporal scales, we conduct ablation experiments with the following STG-STAN variants: “without the node attention mechanism (w/o Att)”, “without the parallel LSTM (w/o PLSTM)”, and “without the node attention mechanism and parallel LSTM (w/o Att-PLSTM)”. These models are trained using multi-region data.

The results of the ablation study are listed in Table 4. Several conclusions from these experimental results are summarized as follows:

- The STG-STAN outperforms all variants, which demonstrates that our proposed method can capture effective information through spatial–temporal modeling. Despite lacking real-world geo-location information, the STG-STAN can achieve accurate predictions by mining potential state transition information from mobile traffic sequences.
- The performance degradation of the STG-STAN without the node attention mechanism indicates that capturing the global correlation of states is critical in the graph structure. After integrating information from neighboring nodes in a GCN, the node attention mechanism is capable of exploiting spatial correlations among nodes in the global graph structure.
- The poor performance of the STG-STAN without parallel LSTM indicates the importance of mining short-term local temporal characteristics. In the case of highly dynamic mobile traffic, the integration of small-scale information can greatly enhance the prediction performance of the model.

## 6. Conclusions

In this paper, we propose a novel STG-STAN for predicting mobile traffic. By representing temporal changes as state transitions, we construct dynamic state graphs to model the traffic’s temporal evolution and capture spatial–temporal correlations. In the STG-STAN, GNNs and a node self-attention mechanism are adopted to fuse transition information and extract global structural information, respectively. Moreover, LSTM networks are developed to capture the evolving information of each node independently in the temporal domain. We also incorporate a parallel LSTM branch to extract small-scale temporal characteristics of mobile traffic. By fusing state spatial–temporal features and parallel LSTM output, the STG-STAN can achieve precise traffic predictions. Our framework improves prediction accuracy by effectively capturing complex spatial–temporal correlations and dependencies between traffic states. Comparative experiments and an ablation study on real-world data validate the effectiveness and robustness of our proposed approach.

## Figures and Tables

**Table 1:** Comparisons of the prediction results. The bolded results indicate the best performance.

Dataset	Traffic	Models	*n* = 1			*n* = 20		
			RMSE	MAE	R2	RMSE	MAE	R2
Milan	SMS	SVR	27.8938	10.8571	0.7896	30.4487	16.2678	0.8409
		RF	**25.4097**	**9.4293**	**0.8253**	27.4147	11.2501	0.868
		LSTM	27.4289	10.9540	0.7943	27.1170	11.2600	0.8706
		CNN-LSTM	-	-	-	32.2569	14.8794	0.8128
		DenseNet	-	-	-	31.7915	18.1345	0.8196
		STG-STAN	26.6621	10.6130	0.8056	**26.8549**	**11.0842**	**0.8725**
	Call	SVR	15.4176	6.3774	**0.9185**	24.6290	13.2826	0.9241
		RF	16.3987	**5.8604**	0.9077	24.7684	9.5313	0.9241
		LSTM	11.8367	8.4304	0.7405	23.5098	9.6391	0.9317
		CNN-LSTM	-	-	-	24.2783	10.7070	0.9287
		DenseNet	-	-	-	29.7551	15.8865	0.8820
		STG-STAN	**10.9830**	7.3755	0.7670	**22.0636**	**9.0541**	**0.9395**
	Internet	SVR	174.0304	59.2985	0.7617	79.4623	58.0813	0.9558
		RF	**110.8407**	**44.1866**	**0.9034**	68.4560	35.8034	0.9685
		LSTM	135.5389	54.2510	0.8555	60.9615	**30.9476**	0.9756
		CNN-LSTM	-	-	-	114.6127	66.4044	0.9142
		DenseNet	-	-	-	276.5915	110.6223	−0.1705
		STG-STAN	152.2309	53.8450	0.8178	**60.3678**	31.1022	**0.9761**
Trentino	SMS	SVR	23.3452	7.8508	0.9093	15.1005	6.1116	0.6828
		RF	**19.8042**	**5.9814**	**0.9347**	11.3088	5.2303	0.8400
		LSTM	24.8605	7.4796	0.8943	10.1354	4.5860	0.8640
		CNN-LSTM	-	-	-	16.1642	7.6263	0.6406
		DenseNet	-	-	-	14.2120	7.5922	0.7342
		STG-STAN	23.2465	7.2252	0.9072	**9.6616**	**4.5008**	**0.8763**
	Call	SVR	**12.9342**	3.5366	**0.9461**	5.9257	2.4553	0.8266
		RF	13.3254	**2.9403**	0.9427	4.7544	1.9579	0.9091
		LSTM	16.7034	4.4159	0.8850	4.4325	2.0017	0.9202
		CNN-LSTM	-	-	-	6.9517	3.2601	0.8260
		DenseNet	-	-	-	7.5137	3.7362	0.7853
		STG-STAN	15.5026	3.9873	0.9010	**4.1654**	**1.9170**	**0.9318**
	Internet	SVR	93.5274	27.1957	0.9080	58.0123	17.8620	0.6548
		RF	64.6846	**19.2679**	**0.9560**	44.9334	17.3566	0.7921
		LSTM	**63.2925**	22.9900	0.8546	38.4114	13.2221	0.8359
		CNN-LSTM	-	-	-	45.2172	21.0177	0.7800
		DenseNet	-	-	-	147.6278	79.1948	−0.7530
		STG-STAN	77.2071	24.5499	0.9347	**38.0165**	**13.1691**	**0.8386**

**Table 2:** Performance of STG-STAN under different numbers of transition states. The bolded results indicate the best performance.

Dataset	*N*	SMS			Call			Internet		
		RMSE	MAE	R2	RMSE	MAE	R2	RMSE	MAE	R2
Milan	5	26.7188	11.1977	0.8745	24.5285	10.0807	0.9261	65.2909	33.2255	0.9721
	10	26.9428	11.1375	0.8718	25.5865	10.0202	0.9202	**60.3678**	**31.1022**	**0.9761**
	15	**26.8549**	**11.0842**	**0.8725**	**22.0636**	**9.0541**	**0.9395**	62.8848	32.8871	0.9724
	20	27.1671	11.3420	0.8696	25.4098	9.8879	0.9210	63.6375	33.0716	0.9736
	30	27.4558	11.2727	0.8669	25.3670	10.3641	0.9213	64.3278	33.6158	0.9728
	40	27.4626	11.5221	0.8667	25.0931	9.9517	0.9233	64.9026	30.8612	0.9723
	50	27.3804	11.4718	0.8675	25.8404	10.3079	0.9186	64.8200	34.2479	0.9726
	60	27.0784	11.4278	0.8711	25.7499	10.1071	0.9190	64.5283	33.9147	0.9728
	70	27.2537	11.5924	0.8693	26.0037	10.1500	0.9174	63.9375	33.8695	0.9732
	80	27.3340	11.4508	0.8688	25.3610	10.2044	0.9216	64.7184	34.5756	0.9726
	90	27.3176	11.7105	0.8685	26.1878	10.7755	0.9165	63.8565	33.5565	0.9735
	100	27.2328	11.5327	0.8690	25.2173	9.9323	0.9224	65.2322	34.4176	0.9722
Trentino	5	10.0422	4.5140	0.8670	4.3159	1.9284	0.9267	**38.0165**	**13.1691**	**0.8386**
	10	10.0360	**4.4825**	0.8682	4.2012	1.9425	0.9312	39.5458	13.5004	0.8231
	15	10.4851	4.5731	0.8589	**4.1654**	**1.9170**	**0.9318**	41.6017	13.7929	0.8005
	20	10.5031	4.7588	0.8628	4.3731	1.9480	0.9220	39.4425	13.2419	0.8323
	30	10.2293	4.5512	0.8617	4.2240	1.9434	0.9288	40.5948	13.2685	0.8317
	40	10.3206	4.5226	0.8574	4.3486	1.9785	0.9228	40.6837	13.5051	0.8116
	50	10.1893	4.5529	0.8628	4.3337	1.9690	0.9252	42.0312	13.7087	0.8079
	60	10.0265	4.5322	0.8684	4.3698	1.9884	0.9247	40.1723	13.4775	0.8182
	70	9.8508	4.4974	0.8723	4.3199	1.9535	0.9266	40.3572	13.4586	0.8180
	80	10.0287	4.5460	0.8677	4.2664	1.9801	0.9288	40.8583	13.5146	0.8140
	90	10.2194	4.5794	0.8625	4.2759	1.9577	0.9299	40.3226	13.4432	0.8162
	100	**9.6616**	4.5008	**0.8763**	4.2954	1.9798	0.9288	40.6897	13.5521	0.8154

**Table 3:** The size of different models and time overhead.

Models	Parameters	Computation Time	
		Training	Inference
SVR	-	40.98 (s)	1.97 (s)
RF	-	28.57 (s)	0.02 (s)
LSTM	21377	4.17 (s/epoch)	0.89 (s)
CNN-LSTM	388692	0.43 (s/epoch)	0.02 (s)
DenseNet	83272	0.92 (s/epoch)	0.05 (s)
STG-STAN	39360	13.39 (s/epoch)	1.85 (s)

**Table 4:** Prediction results in the ablation study. The bolded results indicate the best performance.

Dataset	Traffic	Models	Metrics		
			RMSE	MAE	R2
Milan	SMS	STG-STAN	**26.8549**	**11.0842**	**0.8725**
		w/o Att	26.9117	11.3635	0.8723
		w/o PLSTM	36.0434	22.7099	0.7599
		w/o Att-PLSTM	36.6125	22.4961	0.7511
	Call	STG-STAN	**22.0636**	**9.0541**	**0.9395**
		w/o Att	23.9859	9.7559	0.9286
		w/o PLSTM	30.8017	14.8696	0.8809
		w/o Att-PLSTM	32.1083	15.6051	0.8701
	Internet	STG-STAN	**60.3678**	**31.1022**	**0.9761**
		w/o Att	62.4799	33.5622	0.9742
		PLSTM	147.2356	109.5837	0.8424
		w/o Att-PLSTM	151.5408	113.4919	0.8359
Trentino	SMS	STG-STAN	**9.6616**	**4.5008**	**0.8763**
		w/o Att	10.2407	4.6374	0.8622
		w/o PLSTM	12.8424	5.6754	0.7802
		w/o Att-PLSTM	11.8233	5.4809	0.8185
	Call	STG-STAN	**4.1654**	**1.9170**	**0.9318**
		w/o Att	4.4178	1.9967	0.9234
		w/o PLSTM	6.7825	3.2252	0.8021
		w/o Att-PLSTM	6.2948	3.2013	0.8452
	Internet	STG-STAN	**38.0165**	**13.1691**	**0.8386**
		w/o Att	38.9355	17.6448	0.8314
		PLSTM	63.8827	41.5215	0.6228
		w/o Att-PLSTM	63.9516	41.7513	0.6038

## Data Availability

The data presented in this study are openly available in Harvard Dataverse at 10.1038/sdata.2015.55, reference number [33].

## Abbreviations

A set of historical mobile traffic	*X*
Size of window size	τ
The k-th state transition graph	$G_{k}$
Number of traffic states	*N*
Set of vertices on the *k*-th graph	$V_{k}$
Set of edges on the *k*-th graph	$E_{k}$
Adjacency matrix of the *k*-th graph	$A_{k}$
Number of times when traffic transits from state-*i* to state-*j*	$c_{ij}$
Length of segment-*k*	$l^{k}$
Embeddings of node-*i*	$v_{i}$, $z_{i}$, $h_{i}$, $m_{i}$
Trainable weights	W, b
Attention matrix	α
